# Differences in sedentary time and physical activity among mothers and children using a movement-to-music video program in the home environment: a pilot study

**DOI:** 10.1186/s40064-016-1701-z

**Published:** 2016-01-28

**Authors:** Pipsa P. A. Tuominen, Pauliina Husu, Jani Raitanen, Riitta M. Luoto

**Affiliations:** The UKK Institute for Health Promotion Research, Kaupinpuistonkatu 1, PL 30, 33501 Tampere, Finland; Department of Health Sciences, Faculty of Sport and Health Sciences, University of Jyväskylä, Jyväskylä, Finland; School of Health Sciences, University of Tampere, Tampere, Finland

**Keywords:** Sedentary behavior, Physical activity, Accelerometer, Movement to music, Video, Home environment

## Abstract

Measured objectively, less than a quarter of adults and under half of preschool children in Finland meet the physical activity recommendations. Moreover, higher sedentary time among parents (such as watching television) is associated with higher sedentary time of their children. The study introduces an intervention based on reducing sedentary behavior among mothers and their children. It utilizes a combination of music and exercise via a motivation-targeting movement-to-music video program in the home environment. Data were collected in summer 2014 from Finland’s Pirkanmaa region. Each mother–child pair (*n* = 24, child age: 4–7 years) was assigned to the intervention and control group. Both groups used an accelerometer and completed physical activity diaries for two consecutive weeks (14 days) during waking hours. In addition, the intervention group was instructed to use the movement-to-music video program during the second week. Differences between groups were expected in analysis of sedentary time and physical activity between weeks 1 and 2. The parameters assessed were sedentary time (i.e., lying down or sitting), standing still, and time spent in physical activity. Less sedentary time was revealed in week 2 than in week 1 among both intervention group mothers (56.6 vs. 53.3 %) and for intervention group children (49.5 vs. 46.0 %). The opposite was true of control group mothers (52.1 vs. 52.4 %) and children (46.7 vs. 49.8 %). Within-group differences in mothers’ sedentary time correlated moderately with the children’s sedentary time (Spearman’s *r* = 0.56). All groups exhibited slightly more standing in the second week than in week 1. Both sets of intervention participants also engaged in more light physical activity in week 2, with the opposite evident for the two control sets. In all groups, except the control children, the proportion of moderate to vigorous physical activity was higher in the second week than the first. The use of music and video content together may yield added benefits in efforts to reduce sedentary behavior and increase physical activity among mothers and their children in the home environment.

## Background

Sedentary behavior (SB) is an ever-increasing problem, and many health-related risks, including the risk of type 2 diabetes, obesity, breast cancer, cardiovascular diseases, and all causes of mortality overall, grow as sedentary lifestyles become more widespread (Biswas et al. 2015; Brocklebank et al. 2015; Katzmarzyk et al. 2009; Patel et al. 2010; de Rezende et al. 2014). SB is characterized by low (≤1.5 MET, metabolic equivalent) energy expenditure while one is sitting or lying down during waking hours (Sedentary Behavior Research Network 2012; Tremblay et al. 2010). Physical activity (PA), on the other hand, is associated with reduced risk of metabolic disorders and a lower number of risk factors for other chronic diseases (Cho et al. 2009; Woolf et al. 2008). The PA guidelines for adults recommend at least 150 min of moderate-intensity (3–6 METs) or 75 min of vigorous (≥6 METs) PA or an equivalent combination of aerobic activities every week in sessions of 10 min or more. Also recommended are muscle-strengthening activities for all major muscle groups on two or more days a week (Physical Activity Guidelines Advisory Committee 2008). For children, the PA guidelines recommend 60 min or more of aerobic activity per day, along with muscle- and bone-strengthening activities at least 3 days a week (Physical Activity Guidelines Advisory Committee 2008).

Objective measurements indicate that under a quarter of adults and fewer than half of preschool children meet the PA recommendations (Hnatiuk et al. 2014; Husu et al. 2014; Kettner et al. 2013). Physical environment at home exerts an important influence on children’s SB and PA, and the number of media devices has a positive correlation with screen-based SB (Maitland et al. 2013). Moreover, higher sedentary time of parents (watching television etc.) is associated with greater sedentary time of their children (Jago et al. 2010, Jago et al. 2013). On the other hand, devices used in PA (bicycles, sports equipment, trampolines, etc.) at home may increase light-intensity activity rather than moderate to vigorous physical activity (MVPA), and changes in the physical environment at home (such as limiting TV viewing or engaging in active video gaming instead of passive gaming) have potential for a beneficial influence on children’s SB and PA (Maitland et al. 2013).

Objective measurement with an accelerometer has been recommended for ascertaining SB and PA patterns in combination with diary-based self-reporting (Rosenberg et al. 2014). The accelerometer continuously measures tri-axial acceleration caused by any movement and permits precise assessment of individuals’ SB and PA. Domain-specific SB (such as watching TV, using a computer, or reading) is more reliably reported in diaries than overall SB is. People tend to under-report their SB and over-report vigorous PA when using self-reporting tools as compared with an accelerometer (Dyrstad et al. 2014). In addition, patterns of SB and PA can be characterized in more detail (with differentiation among lying, sitting, standing, and sit-to-stand movements, and in terms of individual sessions of SB and PA and their duration) by means of an accelerometer (Rosenberg et al. 2014).

Finnish women aged 30–39 used the accelerometer, on average, 14 h 20 min (SD 1 h 56 min) per day in the Finnish Health 2011 Study. Here, SB accounted 57 % of this measurement time, standing still (SS) for 18 %, light physical activity (LPA) for 20 %, and MVPA for 5 % (Husu et al. 2014). Corresponding measurement among preschool (i.e., 4–7-year-old) children has not been conducted in Finland. According to Soini et al. (2014a), for most of the childcare day, PA levels and activity types of children are sedentary in nature. However, there remains a research gap in Finland specifically in relation to the PA of children below school age and to the role of families therein (Gråstén et al. 2014). Furthermore, the measurement time has varied greatly in studies conducted among children, and the studies have used diverse measurement tools and analysis algorithms, with definitions for SB and PA too varying between studies (Soini et al. 2014b; Vorwerg et al. 2013; Wen et al. 2010).

Studies on intergenerational transmission of PA and SB have shown that parents play a critical role in their children’s PA and SB (Jago et al. 2013; O’Dwyer et al. 2012; Xu et al. 2014). Early childhood is a good time to promote healthful lifestyle habits (for example sufficiency of daily physical activity), for great benefit to adapt an active lifestyle from young age to adulthood (Bellows et al. 2011). Children from families in which at least one parent has a metabolic disorder (stroke, coronary artery disease, hypertension, or diabetes) tend to be more sedentary than children from families in which there are no metabolic disorders (Chen et al. 2012). Children with active parents seem less likely to be overweight or obese (Erkelenz et al. 2014). In addition, the effects of PA and SB interventions may be stronger for children whose parents meet the PA recommendations, who are active and participate in sports, and who have fewer media devices in the home (O’Dwyer et al. 2012). However, the weak association between the MVPA of 5–6-year-old children and their parents demonstrates that the time for which children are active with their parents is not a major source of PA (Jago et al. 2014). Parents’ support for PA in children seems more important than parents’ acting as a role model (Erkelenz et al. 2014). There is some evidence that SB of children in the form of screen time can be reduced via influencing the screen time of parents (Jago et al. 2013; Xu et al. 2014). Moreover, both PA programs and parental encouragement and support can increase children’s PA (Bellows et al. 2011; Xu et al. 2014).

The benefits found for music in the context of PA and exercise include a positive influence on intensity of PA and level of perceived exertion, alongside extension of workouts, but these have been studied mainly for therapeutic purposes and among athletes (Karageorghis and Priest 2012a, b). Motivational elements of music may improve adherence to exercise plans by increasing users’ motivation for PA (Clark et al. 2015; Inzitari et al. 2009; Karageorghis and Priest 2012a, b). In addition, audiovisual interventions such as viewing video material have a potential effect of shifting attention from internal stimuli (physical sensations) to external cues (auditory and visual stimuli from the music and video content) (Barwood et al. 2009; Hutchinson et al. 2014). Regrettably, the ergogenic effect of combined motivational music and video interventions has seldom been examined. The few studies carried out in this connection point to motivational music combined with video content as an element strengthening the positive effects of audiovisual interventions (Barwood et al. 2009; Hutchinson et al. 2014).

Most work with audiovisual interventions has examined the effects of various (interactive) video games (Adamo et al. 2010; Baranowski et al. 2011, 2012; Paez et al. 2009) or the use of music, television, or videos, in isolation or in combination (Annesi 2001; Bellows et al. 2011; Lin and Lu 2013), highlighting changes in PA or dietary habits, any reduction in perceived exertion, or health outcomes in areas such as cardiorespiratory fitness, body composition, and cholesterol profile. The findings from studies of this nature have proven inconsistent, however. Active video games have been shown to increase light to moderate PA (Biddiss and Irwin 2010; LeBlanc et al. 2013) or overall PA levels (Ni Mhurchu et al. 2008), but additional impact on children’s PA was not detected in naturalistic observation when active and passive video games have been compared (Baranowski et al. 2012). Listening to music CDs while the whole family dance to music as the participating children had learned at school was found useful for improving children’s overall PA level (Bellows et al. 2011). In Dance Revolution study, parents’ and friends’ use of an active video game was found to be associated with children’s initial and sustained participation (Paez et al. 2009).

To our knowledge, previous studies have not considered a combined music and video intervention for mothers and their children that is focused on reducing SB. With this multimedia approach, it might be possible to decrease SB and increase PA. We carried out a pilot study to evaluate the feasibility of a large randomized controlled trial (RCT) examining the effects of a video-based program on mothers’ and children’s PA and SB. That study introduced a combination of music, exercise, and a potentially motivating movement-to-music video program in the home environment.

The core research question is whether there are differences in sedentary time and PA when an accelerometer and movement-to-music video program are used relative to an accelerometer only. The main outcome variables of the pilot study are SB and PA, which were assessed objectively by means of the accelerometer and further examined via PA diaries. We hypothesized that a video program developed for mother–child pairs would be associated with less sedentary time than was only wearing an accelerometer in a comparison of the average times from the first week and the second (intervention) week, and that there would be no differences in MVPA, though the proportion of light-intensity PA would be higher with the movement-to-music video (i.e., in the second week).

## Methods

### Participants

The participants in the pilot study were recruited from the 5-year follow-up cohort from a lifestyle, counseling, and exercise in maternity care project (NELLI). The focus of the NELLI study was on prevention of type 2 diabetes, metabolic syndrome, and obesity among pregnant women. The protocol and methods of the NELLI study have been reported upon in detail previously (Luoto et al. 2010). For our study, 25 mother and child (aged 4–7 years) pairs were invited from Finland’s Pirkanmaa region to take part, and the mothers gave written informed consent. One mother invited to participate declined to take part in the study, making the final number of participants 24 mother–child pairs. Our study was approved by the ethics committee for Pirkanmaa (with ethics license code R14039).

The mother–child pairs were non-randomly assigned to the intervention group (*n* = 11) or control group (*n* = 13). Assignment was performed in 1:1 ratios (the first mother–child pair to the control group and next one to the intervention group) based on the mothers’ interest in taking part in the study. The background of the mothers is presented in Table 1. Both groups were instructed to use a hip-worn accelerometer (Hookie AM20, Traxmeet, Finland) and fill in PA diaries for two consecutive weeks (14 days). In addition, the intervention group received the movement-to-music video program for the second week (days 8–14). Mothers in the intervention group were instructed to exercise in accordance with the video’s directions every other day with their children.

**Table 1:** The mother’s background status

	Intervention group mothers (*n* = 10)	Control group mothers (*n* = 13)
Age (years) (mean, SD)	38.7 (4.3)	38.9 (5.8)
Height (cm) (mean, SD)	169.2 (6.6)	166.5 (5.8)
Weight (kg) (mean, SD)	71.0 (13.7)	70.9 (9.2)^a^
Marital status		
Married	8	11
Cohabiting	2	2
Employment status		
Working	7	7
In part-time work	0	1
On maternity or child-care leave	2	1
Homemaker	1	1
Unemployed	0	2
Unknown	0	1

^a^One mother’s weight was unknown, *n* = 12

The criterion of adequate accelerometer use was met by 23 mothers and 17 children (at least 6 days each week, 10–20 h per day). Because some accelerometer and exercise diary data were missing, one mother–child pair was not considered in the analysis. Furthermore, four children in the intervention group, and two in the control group did not meet the criterion of using the accelerometer at least 4 days each week. Accordingly, for analysis purposes the intervention group consisted of 10 mothers and six children, while the control group was made up of 13 mothers and 11 children. The two groups of mothers did not differ in background characteristics (see Table 1). All children were born in 2008–2009, with the mean age being 5.7 (SD 0.3) years in the intervention group, and 5.5 (SD 0.4) years in the control group. The intervention group consisted three girls and three boys, and the control group had two girls and nine boys.

### The movement-to-music video program

The movement-to-music program was prepared by music-education students and their teachers at Finland’s Sibelius-Academy in a course specifically on children’s music programs. The music in the videos encompasses children’s rock, folk, and Latin music, all performed by a band and singer. The exercises in the videos are based on PA recommendations and include exercises designed to improve or maintain aerobic fitness, muscle strength, balance, and coordination. The material takes the form of three sections, each lasting about 10 min. These could be used individually or all three consecutively. The purpose of the video was to motivate mother and child to exercise together to the beat of the music. Instructions were provided, to allow the mother and child to choose suitable movements for themselves from one to three variations. The details of use of this video-based movement program have been reported upon previously (Tuominen et al. 2015).

### Measurements

Tri-axial accelerometer data were analyzed in raw mode, with the focus being on mean amplitude deviation (MAD) of acceleration (Vähä-Ypyä et al. 2015a). For analysis purposes, SB consisted of lying and sitting down (<1.5 METs). We analyzed standing still (SS < 1.5 METs) and light PA (LPA 1.5–3 METs) separately, and moderate to vigorous PA formed the MVPA category (≥3 METs). Only participants who had used the accelerometer for 10–20 h on each of at least four days in each week were included in the analysis (Herrmann et al. 2013; Matthews et al. 2012; Trost et al. 2005).

As for the PA diary data, participants were asked to record their working hours and the start and end times of PA, such as walking, jogging, running, swimming, biking, gym workouts, and dancing. The mothers were also instructed to assess the perceived exertion involved in their PA with the number 1 (light), 2 (moderate), or 3 (vigorous). Also, the mothers were given diaries for the children, in which they were to record the child’s day-care or preschool time, exercises, and the time spent in PA. Participants could report more than one exercise performance on a given day. The PA types and times reported in the diaries were examined for both mothers and children. Perceived exertion was analyzed for the mothers.

### Statistical analysis

The effects of the intervention were analyzed in line with the intention-to-treat principle through comparison of the differences in the main outcome variables between the intervention and control groups for the first and the second week. Descriptive analysis was used to outline the characteristics of the participants, with tabulation of frequencies (and proportions) or means and standard deviation (SD). The average of the total measurement times for each of the participant groups was used, and the average SB, SS, LPA, and MVPA times as a proportion of the total measurement time were compared between the intervention and control groups. The proportion of measurement time was used because the total measurement time differed between the two weeks and also between the groups. On account of the non-normal distribution of the accelerometer variables, the Wilcoxon signed-rank test was used for repeated measurements (within-group changes) and ordered logistic regression models (also known as proportional odds models) for analyzing the association between the accelerometer variables and the independent groups (intervention vs. control). For the use of ordered logistic regression models, continuous accelerometer variables were examined in tertiles. The models were adjusted for the time for which the accelerometer was worn during the first week and the gender of the participating children, and the assumption of proportional odds (i.e., that regression coefficients are constant across the various levels of the dependent variable) was tested for all models, with the test of parallel lines. A *p* value below 0.05 was considered statistically significant. Spearman’s correlation was used to test the association between mothers and children for changes in sedentary time.

Also, outcomes indicated by the PA diaries—self-reported exercise type, exercise time, and perceived exertion in both the first and the second week—were compared between the intervention and control group. Frequencies of various types of activity were calculated, to describe possible changes between the first and second week in the intervention and control groups, separately.

The data were collected in summer 2014 and analyzed in autumn 2014 to spring 2015. All statistical analyses were conducted with the statistical analysis software package SPSS Statistics (version 22) from IBM.

## Results

### The measurement time

The first set of analyses considered the differences in measurement time. The groups’ time wearing the accelerometer in the first and the second week is shown in Table 2. On average, mothers in the intervention group wore the accelerometers more during the first than the second week. Mothers in the control group used their accelerometer approximately the same amount between the 2 weeks but less than mothers in the intervention group. As for the children, those in the intervention group used the accelerometer more during the first week than in the second, on average, and, overall, children in the control group used it less than children in the intervention group did. The difference in measurement time among children was in parallel to that of mothers.

**Table 2:** Times using the accelerometer during the first and second week

	Intervention group mothers (*n* = 10), time per day (SD)	Control group mothers (*n* = 13), time per day (SD)	Intervention group children (*n* = 6), time per day (SD)	Control group children (*n* = 11), time per day (SD)
Week 1	14 h 29 min (1 h 23 min)	13 h 59 min (1 h 4 min)	13 h 21 min (39 min)	12 h 26 min (25 min)
Week 2	14 h 12 min (1 h 6 min)	13 h 58 min (1 h 12 min)	12 h 59 min (43 min)	12 h 25 min (49 min)

### Sedentary time

Table 3 presents sedentary time as a proportion of the measurement time in weeks 1 and 2. As was hypothesized, both mothers and children in the intervention group were, on average, less sedentary in the second week than in the first. While the SB of the control group participants was expected to remain unchanged, control mothers showed slightly more SB in the second week relative to the first one. In contrast to the other groups, the control children were more sedentary during the second week than in week 1. However, the difference between groups was not statistically significant. Within-group differences were not statistically significant in any of the groups either.

**Table 3:** Differences in sedentary behavior, standing still, and physical activity between weeks 1 and 2

Group	Percentage of measurement time in week 1, mean (SD)	Percentage of measurement time in week 2, mean (SD)	Median of change, percentage points (min., max.)	Wilcoxon test (changes within group), *p* value	Ordered logistic regression, adjusted for time wearing the accelerometer in the first week, estimate (95 % CI) and *p* value
Sedentary time					
Intervention mothers (*n* = 10)	56.6 (8.9)	53.3 (10.0)	−3.9* (−12.9, 10.1)	0.20	−0.40 (−2.02 to 1.22), *p* = 0.63
Control mothers (*n* = 13)	52.1 (7.9)	52.4 (7.4)	−0.8 (−8.8, 19.7)	0.81	
Intervention children (*n* = 6)	49.5 (5.4)	46.0 (5.9)	−3.4 (−8.0, 0.6)	0.075	−1.09 (−2.94 to 0.76), *p* = 0.25
Control children (*n* = 11)	46.7 (6.7)	49.8 (6.5)	+1.7 (−5.9, 14.2)	0.11	
Standing still					
Intervention mothers (*n* = 10)	17.8 (5.6)	19.8 (5.4)	+2.2** (−3.0, 6.0)	0.14	0.63 (−0.94 to 2.19), *p* = 0.43
Control mothers (*n* = 13)	18.0 (6.2)	18.9 (5.7)	+0.8 (−6.7, 5.8)	0.31	
Intervention children (*n* = 6)	9.0 (3.1)	9.2 (3.4)	+0.2 (−2.1, 1.9)	0.75	−0.98 (−2.82 to 0.86), *p* = 0.30
Control children (*n* = 11)	7.9 (1.9)	8.0 (2.0)	+0.1 (−2.4, 1.8)	0.79	
Light physical activity					
Intervention mothers (*n* = 10)	21.6 (3.7)	22.3 (5.8)	+0.6 (−6.5, 6.9)	0.65	0.82 (−0.74 to 2.38), *p* = 0.30
Control mothers (*n* = 13)	25.6 (5.0)	24.0 (3.9)	−0.2 (−16.9, 3.4)	0.55	
Intervention children (*n* = 6)	31.2 (4.6)	33.2 (5.1)	+1.3 (−0.4, 5.3)	*0.046*	0.70 (−0.98 to 2.39), *p* = 0.41
Control children (*n* = 11)	32.5 (4.1)	31.2 (3.6)	−0.4 (−8.6, 3.0)	0.33	
Moderate to vigorous physical activity					
Intervention mothers (*n* = 10)	4.0 (1.5)	4.4 (1.6)	+0.1 (−3.3, 3.3)	0.65	−0.45 (−1.98 to 1.08), *p* = 0.56
Control mothers (*n* = 13)	4.3 (1.8)	4.6 (2.1)	+0.7 (−3.2, 2.6)	0.15	
Intervention children (*n* = 6)	10.4 (2.3)	11.6 (3.4)	+1.7 (−1.0, 2.7)	0.26	1.99 (0.14 to 3.83), *p* *=* *0.035*
Control children (*n* = 11)	12.9 (2.6)	11.1 (2.5)	−1.8 (−8.4, 2.6)	0.060	

Italic values indicate statistical significance at *p* < 0.05

* If +, sedentary time increased, and if −, sedentary time decreased. The latter is a positive change

** If +, SS, LPA or MVPA increased, and if −, it decreased. The first is a positive change

As is shown in Table 3, all groups averaged more SS in the second week than in the first, but neither within-group nor between group differences were statistically significant.

Percentage differences in mothers’ sedentary time correlated positively with percentage differences in children’s sedentary time. Spearman’s correlation for differences in SB between individual mother–child pairs (*n* = 17) was moderate, *r* = 0.56.

### Physical activity as indicated by accelerometer data

It can be seen from the data in Table 3 that the intervention group mothers and intervention group children averaged more LPA during week 2 than week 1, as hypothesized, but the reverse was true for the control (it was expected that overall PA among the control mothers and control children would remain unchanged, as would SB). The within-group difference between the first and second week was statistically significant among the intervention children but not in the other groups. The between groups difference for neither children nor mothers was statistically significant.

Children in the intervention group engaged in more MVPA during the second than in the first week, and more than children in the control group during week 2 (see Table 3). In contrast to the intervention children, the group of control children engaged in less MVPA in the second week than in the first week. The differences between the 2 weeks in MVPA for the two groups of children were in opposite directions, and the difference between groups here was statistically significant. Both sets of mothers showed more MVPA during the second week than in the first. None of the within-group differences were statistically significant, nor were differences between the mother groups.

### Physical activity as reported in the PA diaries

Mothers filled in PA diaries for themselves and the participating child. In all, a completed diary was returned for 22 mothers and 21 children (10 mothers and 10 children belonging to the intervention group and 12 mothers and 11 children in the control group). Adequate information (reported PA type, PA times, and perceived exertion in the first and second week) was obtained from seven mothers and three children in the intervention group and from seven mothers and five children in the control group. Because the quantity of missing data (on time engaging in PA or level of perceived exertion) was considerable, only PA frequencies were taken into account in the analyses.

Mothers in the intervention group reported 48 occasions of PA, of whatever type, in the first week and 47 in the second week. The corresponding numbers among the control group mothers were 45 and 39, respectively. The data showed that the three most popular types of PA among both sets of mother were walking, cycling, and running during the first week. In the second week, the same types were the most popular in the control group, but the intervention group reported walking and cycling also, along with gym visits. Intervention group children were reported to be physically active 24 times during the first and 31 times during the second week. The corresponding numbers for the control children were 28 and 17. The most popular types of activity for children in both groups in the first week were biking, swimming, and soccer, in decreasing order of popularity. In the second week, the most popular types of PA in the intervention group were still biking and soccer, but movement-to-music with the video program was mentioned too. For the control group in week 2, biking, swimming, and walking were at the top of the list, with soccer and other exercise also mentioned.

## Discussion

The pilot study analyzed sedentary time and PA among mothers and children who were using movement-to-music video program at home. This study combined practical implementation of PA measurement with study of motivational music programs. Since the use of music and video material together could aid in reducing SB and increasing PA among those mothers who have difficulties in exercising outside the home while tending to young children, there is a possibility of such interventions producing health benefits through PA in the home. Therefore, the study had potential to reveal one way of decreasing SB and thereby preventing otherwise forthcoming health problems.

The first hypothesis was that the use of the movement-to-music videos within the intervention group would be associated with a decrease in sedentary time while sedentary time in the control groups remained at the same level. The results indicated that there was a trend for less sedentary time in each set of intervention group participants (mothers and children), on average, as a proportion of measurement time in the second week as compared to the first week. This was marginally the case in the group of control mothers also. Differences in SB between the two groups of children were not statistically significant either, although the control children seemed to be more sedentary than the intervention ones during the second week. Without adjustment for the differences in measurement time, the median positive difference (i.e., reduction in SB) between the 2 weeks was around 22 min per day for both sets of mothers and among the intervention group children. This time is fully consistent with findings presented in a systematic review by Martin et al. (2015). In addition, the moderate correlation for differences in sedentary time shows that higher sedentary time of mothers was associated with higher sedentary time of their children. This result too is in agreement with earlier studies (Jago et al. 2010).

Furthermore, accelerometer data showed that, as predicted, both mothers and children in the intervention group showed a trend towards engaging more LPA during the second week than during the first week, although the within group differences were statistically significant only for children. The opposite trend was true for the control mothers and control children. All four sets displayed slightly more SS in the second week as compared to the first. Even though the difference between weeks was small, these findings support the assertion that the intervention may yield positive changes in sedentary time. It has previously been shown that use of active video games is associated with an increase in light to moderate intensity PA (LeBlanc et al. 2013), and it is possible that the movement-to-music video program has the same effect.

The second hypothesis was that there would be no differences in MVPA. However, specifically in the groups of children there were greater differences than expected. Both sets of mothers and the intervention group children performed more MVPA in the second week relative to the first, while the reverse was true for the control children. Within-group differences were not statistically significant, nor were differences between the groups of mothers. However, differences between the two sets of children were statistically significant. The video program’s instructions on exercise frequency and the level of intensity that moving to the program entailed were not very demanding, so we did not expect these differences, although some studies have shown that a motivational music and video intervention might improve high-intensity exercise performance (Barwood et al. 2009). It is possible that music and video content in combination elicit the most positive responses no matter the exercise intensity (Barwood et al. 2009; Hutchinson et al. 2014).

Any conclusions based on the results must take the measurement time into consideration. When accelerometers are used during waking hours only, it is impossible to predict how long each day the participants are going to use them. Differences in measurement time between weeks and between groups were taken into account through analysis based on percentages of the measurement time. The measurement time and the SB, SS, LPA, and MVPA proportions found in our study are in line with the corresponding figures from the Finnish Health 2011 Study (Husu et al. 2014).

The diary data indicate that children in the intervention group exercised more frequently during the second week than children in the control group did. In addition, the reduction in the amount of mothers’ PA was less in the intervention group than in the control group. These findings are consistent with the accelerometer data from the pilot study. However, it is noteworthy that people have a tendency to report more MVPA and less sedentary time in diaries than reflected in the accelerometer measurements (Dyrstad et al. 2014).

### Strengths and limitations

The study is unique in implementing an intervention for mothers and their children at the same time and being aimed at reducing SB in the home environment. The primary strength of the study lies in its objective measurement of SB and PA. Furthermore, the study utilized a new method of analyzing accelerometer data that has been proven to be valid (Aittasalo et al. 2015; Vähä-Ypyä et al. 2015b). The majority of the mothers used the accelerometer and filled in the PA diary as instructed, although there were sometimes missing data, specifically the time used for exercising and the perceived exertion during PA. Also, there were challenges in obtain enough accelerometer data from the children, especially in the intervention group. Also, there is a limitation linked to the nature of hip-worn accelerometers, which are likely to underestimate stationary movements (such as exercising in a gym or riding a bicycle) and unable to detect water-based activities. Nonetheless, accelerometers are the sensitive device to detect overall PA and SB (Vähä-Ypyä et al. 2015a), which was the main focus of the study. These challenges notwithstanding, examining and implementing an intervention for two generations at the same time and using a movement-to-music video program are clear strengths of the study.

The main limitations are the study’s small sample size and short follow-up. In the pilot setting, we detected only minor differences in SB and PA between the first and second week. It is reasonable to assume that a larger number of participants and a longer follow-up time would yield more reliable results. With these conditions, it should also be possible to estimate whether changes in SB and PA occur in the long run and thereby judge the intervention’s effectiveness in relation to the PA recommendations. The need for a study with a larger sample size and longer follow-up time will be in met in ongoing Moving Sound study (Tuominen et al. 2015), registered at ClinicalTrials.gov with ID NCT02270138.

## Conclusions

This pilot study provided information on the effectiveness of movement-to-music video material in influencing the level of SB and PA among mothers and their children. The use of music and video content together may hold additional force in reducing SB and increasing PA. For those mothers and children who have difficulties in exercising outside the home, a movement-to-music video program might represent an important element of motivation to be physically active.

## Abbreviations

LPA: light physical activity
MAD: mean amplitude deviation
MET: metabolic equivalent
MVPA: moderate to vigorous physical activity
NELLI: lifestyle modification, counseling, and exercise in maternity care 5-year follow-up study
PA: physical activity
SB: sedentary behavior
SS: standing still
